# A Spiral in the Heart: Mitral Valve Endocarditis with Unusual Vegetation Shape Potentially Affecting Effectiveness of Antibiotic Therapy

**DOI:** 10.1155/2015/483067

**Published:** 2015-10-25

**Authors:** Veronica Fibbi, Piercarlo Ballo, Alessandro Abbondanti, Marco Nannini, Antonio Fazi

**Affiliations:** Cardiology Unit, S. Maria Annunziata Hospital, 50012 Florence, Italy

## Abstract

We report an unusual case of infective endocarditis (IE) in an 88-year-old woman, occurring on a prolapsing mitral valve and characterized by an atypical vegetation shape resembling a spiral-like appearance. After the patient refused surgical correction, persistent IE despite prolonged antibiotic therapy was observed, resulting in an ischemic stroke probably secondary to septic embolus. The importance of vegetation shape in the management of patients with IE was classically related to the increased risk of embolization associated with pedunculated, irregular, and multilobed masses. We hypothesize that the unusual spiral-like vegetation shape in our patient may have favored IE persistence by two mechanisms, namely, a decrease of the exposed vegetation surface with creation of an internal core where the penetration of antimicrobial agents was obstacled and the creation of blood turbulence within the vegetation preventing a prolonged contact with circulating antibiotics. These considerations suggest that vegetation shape might be considered of importance in patients with IE not only because of its classical association with embolization risk, but also because of its potential effect on the efficacy of antibiotic therapy.

## 1. Introduction

Infective endocarditis (IE) is a major cause of morbidity and mortality worldwide, and stroke secondary to septic embolus is a major and potentially life-threatening complication of left-sided IE [1]. Although early diagnosis, prompt starting of adequate antibiotic therapy, and surgery when appropriate are the cornerstones of successful IE treatment, it is known that the ability of antimicrobial agents to penetrate into the vegetations is a key factor to determine the clinical outcome of IE patients [2]. We report a case of persistent IE followed by ischemic stroke, where an atypical vegetation shape might have contributed to reduce the penetration of the antimicrobial agents, favouring IE persistence and septic embolization.

## 2. Case Presentation

**Figure 1:**
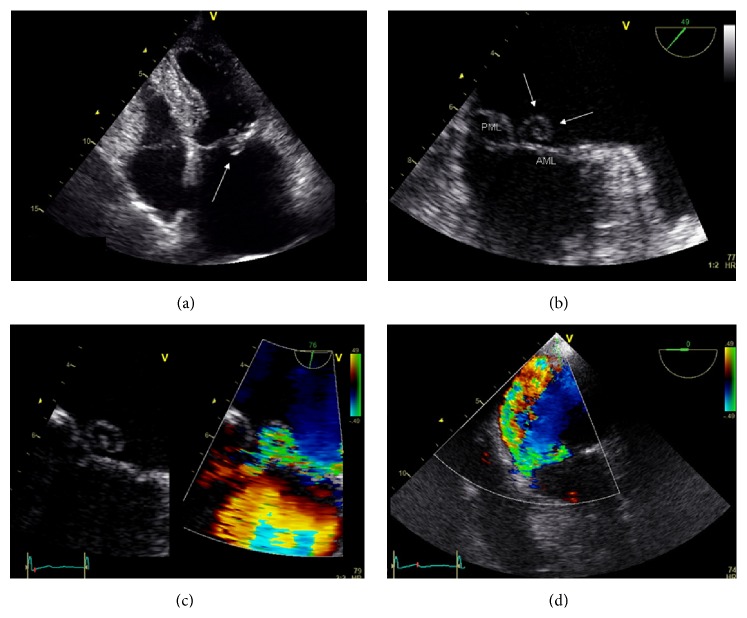
(a) Transthoracic echocardiography showing a rounded-shaped mass attached to a prolapsing posterior mitral leaflet. (b) Transesophageal echocardiography revealing a spiral-like appearance of the mass. (c) Color Doppler image showing high turbulence within the vegetation. (d) Color-Doppler image showing severe mitral regurgitation.

A 88-year-old woman with history of hypertension presented with persistent fever, recent-onset dyspnea, and new-onset systolic murmur. One week earlier, she had started therapy with amoxicillin/clavulanate because of a dental abscess. Clinical examination showed signs of heart failure and a loud systolic murmur at the mitral area. Transthoracic echocardiography (TTE) showed an inhomogeneous, rounded-shaped, mobile mass attached to a prolapsing posterior mitral leaflet (Figure 1(a), Movie 1 in Supplementary Material available online at http://dx.doi.org/10.1155/2015/483067) with severe mitral regurgitation (MR), suggesting infective endocarditis (IE). Blood cultures were taken despite the fact that the patient was under antibiotic therapy, and treatment with ceftriaxone, vancomycin, gentamicin, furosemide, and atenolol was started. Transesophageal echocardiography (TEE) confirmed the presence of a mobile endocarditic vegetation (9 × 8 mm) attached to the atrial side of the prolapsing P2-P3 scallops, revealing its rounded appearance to be caused by an unusual, regular spiral-like shape (Figure 1(b), Movie 2). High turbulence within the vegetation (Figure 1(c), Movie 3) with severe MR (Figure 1(d), Movie 4) was also observed. Blood cultures yielded negative results. On day 9 from admission, because of persistent fever and worsening renal function despite the improvement in signs of heart failure, vancomycin was substituted with teicoplanin. On day 20, a new TEE showed persistence of IE with no changes in vegetation size. Surgery was advised, but the patient refused the intervention. She was discharged under teicoplanin, furosemide, atenolol, and aspirin, with the recommendation to complete 6 weeks of intravenous antibiotic treatment daily at our hospital. A clinical, biohumoral, and instrumental follow-up was planned. Five weeks after discharge, signs and symptoms of heart failure were considerably improved, but the vegetation was still present at TTE. Ten weeks later, the patient was urgently rehospitalized because of ischemic stroke. TTE showed a reduction in the size of the mass with persistence of severe MR, and there was no evidence of carotid or vertebral artery disease. Despite clinical improvement after therapy and rehabilitation, neurological recovery was only partial because of persistent disequilibrium. On day 13, the patient was transferred to a hospice for long-term care.

## 3. Discussion

Echocardiography plays a key role in the clinical management of patients with IE, providing valuable information for IE diagnosis, identification of complications such as heart failure or abscesses, indications to surgery, and prognostic stratification [3, 4]. In particular, careful evaluation of vegetation characteristics in left-sided IE is of major importance to assess the risk of systemic embolization and related clinical events, which range from incidental findings on advanced imaging to potentially devastating events such as stroke and brain abscess [5–7]. According to the current guidelines, patients with large vegetations (>10 mm) should be referred for surgery in the presence of one or more embolic events despite appropriate antibiotic therapy, or if predictors of complicated course (e.g., heart failure or persistent infection) are present [8]. However, predicting the risk of embolism in the individual patient remains difficult [9]. Though the size of the vegetation seems to be the strongest independent predictor of a new embolic event in patients with IE, several other factors are involved, including mobility, location, echogenicity, and shape of the vegetations, potential changes in vegetation size during antibiotic therapy, underlying pathogen, multivalvular IE, and biological markers [5, 10–13]. In this context, the importance of vegetation shape classically hinged on the evidence that pedunculated, irregular, and multilobed vegetations may lead to relatively high risk of embolization. However, less attention was given to the potential role of vegetation shape in affecting the efficacy of antimicrobial treatment.

In this report, we describe a case of IE in which an atypical vegetation shape, characterized by a spiral-like appearance, might potentially have contributed to the persistence of valve infection despite prolonged antibiotic therapy, resulting in a cerebral stroke probably secondary to septic embolus.

It is known that vegetations are mostly composed of layers of fibrin and clusters of buried, slow-growing, and dormant bacteria that tend to escape drug-induced killing by tolerance phenomena [14, 15], so that the efficacy of antibiotic treatment in patients with IE is strictly affected by the ability of the antimicrobial agent to penetrate into the depths of the vegetations, where the microcolonies of the pathogen are located [16, 17]. We hypothesize that the unusual spiral-like shape of the vegetation in our patient may have favored IE persistence by two mechanisms: (1) reduction of the vegetation surface exposed to the blood, with creation of a relatively protected core where the penetration of antimicrobial agents was obstacled and (2) high blood turbulence within the vegetation during the systolic regurgitation, which may have further prevented a prolonged contact of vegetation surface with circulating antibiotics. These considerations suggest that, in the management of patients with IE, the shape of a vegetation might be considered not only a determinant of embolization risk, but also a factor potentially affecting the efficacy of antibiotic therapy.

## Supplementary Material

Movie 1: Transthoracic ecochardiography . Apical four chambers view showing a mass attached to a prolapsing posterior mitral leaflet.
Movie 2: Transesophageal echocardiography. Mid-esophageal view showing a spiral-like endocarditic vegetation.
Movie 3: Transesophageal echocardiography. Color Doppler imaging showing high turbulence within the vegetation.
Movie 4: Transesophageal echocardiography. Color Doppler imaging showing severe mitral regurgitation.

## References

[1] Thuny F., Avierinos J.-F., Tribouilloy C. (2007). Impact of cerebrovascular complications on mortality and neurologic outcome during infective endocarditis: a prospective multicentre study. *European Heart Journal*.

[2] Besnier J. M., Choutet P. (1995). Medical treatment of infective endocarditis: general principles. *European Heart Journal*.

[3] Thuny F., Grisoli D., Cautela J., Riberi A., Raoult D., Habib G. (2014). Infective endocarditis: prevention, diagnosis, and management. *Canadian Journal of Cardiology*.

[4] Thuny F., Di salvo G., Belliard O. (2005). Risk of embolism and death in infective endocarditis: prognostic value of echocardiography: a prospective multicenter study. *Circulation*.

[5] Vilacosta I., Graupner C., SanRomán J. (2002). Risk of embolization after institution of antibiotic therapy for infective endocarditis. *Journal of the American College of Cardiology*.

[6] Tsirka V., Maletic J., Ioannidis P., Karacostas D. (2012). Aortic graft infection as a cause of multiple brain infarcts. *Case Reports in Cardiology*.

[7] Stawicki S. P., Firstenberg M. S., Lyaker M. R. (2013). Septic embolism in the intensive care unit. *International Journal of Critical Illness and Injury Science*.

[8] Habib G., Hoen B., Tornos P. (2009). Guidelines on the prevention, diagnosis, and treatment of infective endocarditis (new version 2009): the task force on the prevention, diagnosis, and treatment of infective endocarditis of the European Society of Cardiology (ESC). Endorsed by the European Society of Clinical Microbiology and Infectious Diseases (ESCMID) and the International Society of Chemotherapy (ISC) for Infection and Cancer. *European Heart Journal*.

[9] Castiñeira-Busto M., Abu-Assi E., Martínez-Monzonis A., Peña-Gil C., Raposeiras-Roubin S., González-Juanatey J. R. (2015). Predicting the risk of systemic septic embolism in patients with infective endocarditis. *Revista Espanola de Cardiologia*.

[10] Okonta K. E., Adamu Y. B. (2012). What size of vegetation is an indication for surgery in endocarditis?. *Interactive Cardiovascular and Thoracic Surgery*.

[11] Di Salvo G., Habib G., Pergola V. (2001). Echocardiography predicts embolic events in infective endocarditis. *Journal of the American College of Cardiology*.

[12] Rizzi M., Ravasio V., Carobbio A. (2014). Predicting the occurrence of embolic events: an analysis of 1456 episodes of infective endocarditis from the Italian Study on Endocarditis (SEI). *BMC Infectious Diseases*.

[13] Hill E. E., Herijgers P., Claus P., Vanderschueren S., Peetermans W. E., Herregods M.-C. (2008). Clinical and echocardiographic risk factors for embolism and mortality in infective endocarditis. *European Journal of Clinical Microbiology and Infectious Diseases*.

[14] Collins J. A., Zhang Y., Burke A. P. (2014). Pathologic findings in native infective endocarditis. *Pathology Research and Practice*.

[15] Cabell C. H., Abrutyn E., Karchmer A. W. (2003). Cardiology patient page. Bacterial endocarditis: the disease, treatment, and prevention. *Circulation*.

[16] Schierholz J. M., Beuth J., Pulverer G. (2000). ‘Difficult to treat infections’ pharmacokinetic and pharmacodynamic factors—a review. *Acta Microbiologica et Immunologica Hungarica*.

[17] Senoune A., Benghalem A., Erdogan K., El Brouzi A., Vergnaud J. M. (1994). Intravegetation antimicrobial distribution in endocarditis: a numerical model and establishment of the conditions for an in-vitro test. *International Journal of Bio-Medical Computing*.

